# A Modern Flexitarian Dietary Intervention Incorporating Web-Based Nutrition Education in Healthy Young Adults: Protocol for a Randomized Controlled Trial

**DOI:** 10.2196/30909

**Published:** 2021-12-21

**Authors:** Andrea Braakhuis, Nicola Gillies, Anna Worthington, Scott Knowles, Tamlin Conner, Rajshri Roy, Toan Pham, Emma Bermingham, David Cameron-Smith

**Affiliations:** 1 Discipline of Nutrition Faculty of Medical and Health Science The University of Auckland Auckland New Zealand; 2 AgResearch Ltd Smart Foods Innovation Centre of Excellence Te Ohu Rangahau Kai Palmerston North New Zealand; 3 Department of Psychology University of Otago Dunedin New Zealand; 4 Agency for Science, Technology and Research Singapore Institute for Clinical Sciences Singapore Singapore

**Keywords:** protein, meat, vegetarian, eating patterns, diet, nutrition, dietary restrictions, biomarkers, health, well-being, macronutrients, micronutrients

## Abstract

**Background:**

The trend of flexitarian eating patterns is on the rise, with young adults among the biggest adopters claiming health and environmental reasons to reduce red meat intake. Nutrient-dense meat and animal products are often the lynchpin of these diets, even when consumed only occasionally and in moderate amounts. Red meat provides forms and concentrations of essential proteins, lipids, and micronutrients that are scarce in exclusively vegetarian regimens.

**Objective:**

The aim of this study is to consider the effects of moderate consumption of lean red meat as part of an otherwise vegetarian balanced diet and its impact on biomarkers of sustained health and well-being.

**Methods:**

A cohort of healthy, young (20-34 years old, n=80) male and female participants will take part in a 2-arm, parallel randomized controlled trial (RCT) for a duration of 12 weeks, with a 3-month posttrial follow-up. The trial will commence with a 2-week assessment period followed by allocation to the intervention arms. The intervention will include the consumption of red meat or meat alternatives 3 times per week for 10 weeks. Blood samples of the participants will be collected to measure changes in erythrocyte fatty acid distribution, circulating amino acids, neurotransmitters, markers of mineral status, and inflammatory markers. Questionnaires to assess well-being and mental health will be undertaken every 2 weeks. Body composition, physical function, and blood parameters will be assessed at allocation (t_0_), week 5 into the intervention (t_5_), and post intervention (t_10_).

**Results:**

The protocol has been developed using the SPIRIT (Standard Protocol Items: Recommendations for Interventional Trials) checklist and the outcomes will be reported in accordance with the CONSORT (Consolidated Standards of Reporting Trials) guidelines. The trial was approved by the New Zealand Ministry of Health’s Health and Disability Ethics Committees (protocol 20/STH/157). The results of this study will be communicated via publication.

**Conclusions:**

To our knowledge, this is the first RCT investigating the overarching health consequences of consuming pasture-fed red meat or no meat as part of a healthy diet.

**Trial Registration:**

ClinicalTrials.gov NCT04869163; https://clinicaltrials.gov/ct2/show/NCT04869163

**International Registered Report Identifier (IRRID):**

PRR1-10.2196/30909

## Introduction

Although not always labeled so, “flexitarianism” is the default lifestyle for much of the world, whereby plant-derived foods provide the bulk of a person’s calories [1,2]. Nutrient-dense meat and animal products can be an important pillar of these diets, even when consumed only occasionally and in moderate amounts [1]. They provide high-quality protein, essential fatty acids, and micronutrients that are limited in concentration or bioavailability in exclusively plant-based diets, notably including vitamin B_12_, iron, zinc, and long-chain omega-3 fatty acids (LCn3) [3]. However, production of meat is resource-intensive and widely censured for its environmental impacts [4]. As a consequence, the trend of meat-rich dietary patterns in Western consumers may not be sustainable.

The role of meat in the diet is under debate, not only from sustainability and ethical considerations, but also from a health perspective based on evidence that habitual consumption increases the risk of some metabolic diseases and cancers [5]. However, much of that research has been retrospective and reliant on food recall, which is known to yield equivocal results and oversimplified recommendations (eg, not distinguishing between intact red meats and processed, cured, or preserved meats) [6]. Randomized trials have more control over diet composition but have tended to feed relatively high levels of meat [5,7] that exceed those recommended by the World Cancer Research Fund guidelines [8]. These trials, which do not consider the potential of moderation, are yielding increasingly contradictory outcomes.

Individuals might choose to avoid or limit meat intake entirely for reasons of personal preference, ethical stance, culture, religion, or health [9]. Vegetarianism has been reported to reduce mortality on account of diverse protective effects [10]. However, research results are inconsistent, the underlying reason for which is yet undefined and the observed health benefits may be confounded by “lifestyle” factors associated with socioeconomic status, such as adequate levels of physical activity [5,11-13]. The association between meat eating and disease tends to be higher in North American [14] than in Asian cohort studies [15], indicating the presence of lifestyle bias, a meat dose effect, and nutritional differences in the meat produced in certain regions [2,16].

Diet is more than just adequate nourishment. Eating well can be a pleasure and a challenge that affects many aspects of well-being and quality of life. A Scandinavian study that compared teenage omnivores versus low-meat consumers found that symptoms of depression and anxiety were considerably worse among the latter [17]. This may have been related to the absence of meat or because the adolescents adhering to the low-meat diets did not also follow other typical lifestyle health choices. A broader review of the psychological health of omnivores and meat-abstainers also found that those who avoided meat consumption had significantly higher rates or risk of depression, anxiety, or self-harm behaviors [18]. The components of red meat that may be beneficial for mental health include fatty acids, phospholipids, cholesterol, niacin, vitamin B_6_, and vitamin B_12_, while saturated fat is considered detrimental to cognitive function [19]. In particular, the essential LCn3 are valued for their roles including lipid-lowering properties, mitigation of platelet aggregation and inflammation, and improvements in cognition and mood [20]. In the context of Western diets where seafood is a minor constituent, the contribution of red meat to total LCn3 intake can be substantial [21].

Evidence suggests that young adults are the largest age group who adopted a flexitarian diet [2,16]. Despite the long-lasting impacts of diet during young adulthood, this is an age group not frequently researched in the meat and nonmeat consumption literature, particularly those focusing on their subjective experience to the diet [2]. To our knowledge, no randomized controlled trial (RCT) has investigated aspects of physiological and psychological well-being concurrently, nor in young adults.

The objective of this trial is to compare the physiological and psychological effects of consuming moderate amounts of pasture-raised lean red meat or vegetarian analogues in the context of a balanced diet for 10 weeks. We will measure changes in markers of nutritional status and indices of longer-term health and mental well-being, with the hypothesis that lean red meat will confer benefits related to the presence, concentration, and bioavailability of nutrients which are typically not matched in vegetarian analogues [7,19,22]. The study is part of a larger program to understand the human and environmental implications of consuming pasture-raised (ie, grass-fed) beef and lamb.

## Methods

### Study Design and Setting

The study is a parallel RCT comprising a 2-week preintervention assessment, a 10-week intervention period, and a 22-week posttrial follow‑up. Participants will all be maintained on a balanced vegetarian basal diet and be randomized to also consume either red meat (“Flexitarian” arm) or a meat substitute (“Vegetarian” arm). The meat will be pasture-raised beef and lamb butchered and packaged in accordance with our specifications in New Zealand, while the meat substitute will be commercially available plant-based products (Multimedia Appendix 1). The SPIRIT guidelines were followed when designing the research [23].

Recognizing that there are many interpretations of the terms, “vegetarian” and “flexitarian,” in this study we define vegetarian as ovo-lacto vegetarian and flexitarian as “a vegetarian diet with moderate amounts of red meat.” Both arms can consume eggs and dairy products, but not chicken, pork or fish, and no red meat other than that supplied by the researchers.

The study is designed around pairs of young adult consumers who share meals and are therefore likely to engage over the values and challenges of developing a healthy lifestyle. Couples/household units (see *Recruitment*) will be randomized to the same intervention arm. Depending on their allocation, the couple will receive regular allotments of either frozen red meat or meat-analogue, which will be sufficient to provide 3 meals (approximately 350-500 g of cooked meat or vegetarian alternative per person per week) [8,24,25]. We will evaluate changes in objective and self-assessed subjective health variables between the 2 intervention arms. These include physical function, body composition, metabolic health biomarkers, and psychological well-being.

### Recruitment

We will recruit pairs of individuals (spouses, partners, or companions) who cohabitate and typically share evening meals. Participants will be GenZ (18-23 years old) and millennial (23-34 years old) individuals. This population demographic has the greatest variation in meat intake [8]. Recruitment will be advertised with posters placed around the University of Auckland and using social media websites and tools.

Potential participants will meet with researchers responsible for recruitment in person, where eligibility will be confirmed, and details of the study will be discussed with opportunity for questions to be clarified. At this in-person screening visit, participants will provide written informed consent (Multimedia Appendix 2).

### Eligibility Criteria

All participants are required to be considered omnivores if, in the last 2 months, they consumed at least 2-3 meals per week which included meat of any description (red or white fleshed meat, including fish). They must be willing to consume both red meat and meat analogues for the purposes of the trial. Those with chronic health conditions, obesity (BMI≥30 kg/m^2^), hyperlipidemia, disordered eating patterns, history of anosmia and ageusia (issues with smell and taste), use of medications (except for occasional nonsteroidal anti-inflammatory drugs and antihistamines), or recreational drugs, or those who smoke tobacco will be excluded from participating. Potential participants who use dietary supplements were asked to abstain for the month prior to the beginning of the study. Participants must own a mobile phone with a camera and be proficient with using Facebook and Facebook messenger. Women must confirm they are neither pregnant nor intending to become pregnant during the trial.

Participants will complete a web-based eligibility screening, which includes the Three-Factor Eating Questionnaire (TFEQ-R18), a Self-Efficacy questionnaire, a Food Frequency questionnaire, and a health survey. Participants with a TFEQ score greater than 75% will be excluded on the basis that their perception of food is potentially influenced by underlying psychological issues or that they demonstrate disordered eating patterns. Given the routine monitoring of food intake and subjective experience to food required in this research, this cut-off was deemed clinically relevant for the purpose of this study by the research team, including psychologists and registered dietitians [26]. Participants must own a mobile phone with a camera. Women must confirm they are not pregnant, nor intending to become pregnant during the trial.

Participants will be monitored for their adherence to the study guidelines through the dietary recording smartphone app *Easy Diet Diary* and were prompted for adherence via emails, social media, and SMS text messages. They will have the right to withdraw from the study at any time without any explanation. The principal investigator (PI) will have the right to discontinue participants’ involvement if they become ineligible or when any significant protocol deviations occur in the study. The data of participants who withdraw will be retained and might be used in exploratory analyses, unless the participant requests data to be deleted.

### Sample Size Calculation

The primary biomarker for calculating the sample size is the concentrations of LCn3 in erythrocyte membranes, and a sample size of 63 will provide sufficient power to detect changes in fatty acids, which might be due to the intervention. Published data indicate a change of 3.01 µg/mL (SD 1.1 µg/mL) in erythrocyte fatty acid composition (20:5) following a 2-week dietary intervention and crossover meat trial among young adults [27-29]. The Cohen effect size of 0.2 (small) of the variability in erythrocyte fatty acids was used to consider the smallest worthwhile effect. To allow for dropouts, a total of 80 participants will be recruited.

### Randomization and Blinding

The 80 participants (self-organized as 40 couples/household units) will be randomized evenly to the vegetarian or flexitarian arm, with a random allocation sequence (1:1 ratio) generated using Random.org. Researchers responsible for participant recruitment will be blinded to allocation until participants begin their 2-week lead-in period. Owing to the nature/form of the food provided, participants will not be blinded to their intervention; nonetheless, they will not be aware of this until the intervention begins.

Couples will be further organized into “teams,” which are groupings of 5 couples who consume the same diet. There will be 4 such teams per intervention arm. A team serves as another layer of structure and mutual support during the intervention period. Team members will engage virtually through social media, which was explained to participants during recruitment.

### Ethics and Dissemination

The designed intervention has been assessed by a nutrition expert panel. The trial was approved by the New Zealand Ministry of Health’s Health and Disability Ethics Committees (protocol 20/STH/157). The investigators will ensure that this study is conducted in accordance with the principles of the Declaration of Helsinki, with relevant institutional regulations. Results arising from this study will be submitted for publication in scientific journals and presented at meetings. Authorship will be determined in accordance with the guidelines of the International Committee of Medical Journal Editors.

### Study Overview

**Figure 1:**
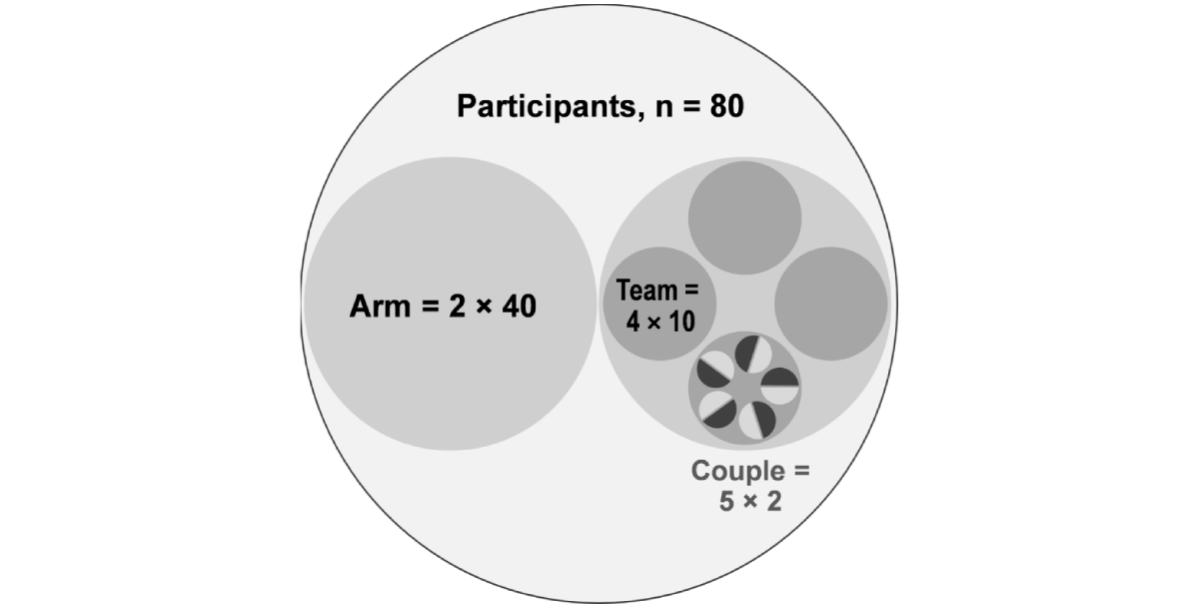
Participation grouping within the 2 intervention arms.

Each participant will be coupled with another participant within a team in each arm (Figure 1). The trial commences with a 2-week preintervention period when participants consume their usual diet. Following allocation, couples will begin to receive allotments of either meat or the meat alternative (3 meals per week) and weekly vegetarian meal kits plus (additional 3 meals per week). The dietary intervention continues for 10 weeks.

At intervals during the trial, participants will take part in assessments of nutritional status and mental well-being in accordance with the schedule shown in Table 1. A follow-up evaluation (on the internet or in person) at week 22 will conclude the study.

Participants will visit the University of Auckland Clinical Research Centre and a blood testing facility in Auckland on three occasions: at baseline (t_0_), midway through intervention at week 5 (t_5_), and at the end of intervention at week 10 (t_10_). A blood sample will be collected from an antecubital vein by a trained phlebotomist following an overnight fast. Body weight, blood pressure, grip strength, and body composition will be measured as described below. Participants will wear a wristband accelerometer for the duration of the study to estimate their level of physical activity and sleep patterns. All participants will complete the suite of mental health questionnaires on up to 7 occasions either on the internet or in person, which takes approximately 15 minutes. From t_0_ to t_10_, participants will record their dietary intake using Easy Diet Diary. Two members of the research team will be responsible for participant recruitment, clinic visit assessments, and nutrition education delivery via the web-based platform. Participants will also be in contact with technical staff at the blood testing facility.

**Table 1:** Schedule of enrolment, interventions, and assessments.

		Study period								
		Enrollment	Preintervention	Intervention						Close-out
Timepoint (week)			–t_2_	t_0_	t_2.5_	t_5_	t_7.5_	t_10_	t_22_	
**Enrollment**										
	Eligibility screening	X								
	Informed consent	X								
	Allocation		X							
**Interventions**										
	Vegetarian^a^			X	X	X	X	X	X	
	Flexitarian with pasture-raised beef and lamb^a^			X	X	X	X	X	X	
**Assessments**										
	Easy Diet Diary monitoring^b^		X	X	X	X	X	X	X	
	Activity/sleep monitor^b^		X	X	X	X	X	X	X	
	MFSI-SF^c^/WHO-5^d^/ PES^e^/DASS‑21^f^ questionnaires		X	X	X	X	X	X	X	
	University of Otago Food Frequency Questionnaire		X			X		X	X	
	Body composition (dual x-ray absorptiometry)			X				X		
	Grip strength/blood pressure/weight			X		X		X		
	Erythrocyte/plasma fatty acids			X		X		X		
	Inflammatory markers			X		X		X		
	Minerals (Fe and Zn)			X		X		X		
	Vitamins (A, D, E, and K)			X		X		X		
	Insulin/glucose/high-density lipoprotein cholesterol/low-density lipoprotein cholesterol			X		X		X		
	Amino acids and 1-carbon nutrients/metabolites			X		X		X		
	Neurotransmitters			X		X		X		
	Self-efficacy questionnaire	X								
	End of intervention survey							X	X	

^a^Continuous monitoring from t_0_.

^b^Continuous monitoring from –t_2_.

^c^MFSI-SF: Multidimensional Fatigue Symptom Inventory-Short Form.

^d^WHO-5: World Health Organization–Five Well-Being Index.

^e^PES: Positive Eating Scale.

^f^DASS-21: Depression Anxiety Stress Scales-Short Form.

### Dietary Intervention

Household units will receive regular deliveries of either red meat or vegetarian analogues sufficient for 3 evening meals per week. The quantity of meat will provide 350-500 g cooked weight per person per week, which conforms to the latest international recommendations for maximum intake [8] and the reported typical frequencies of red meat consumption [24,25]. The meat alternatives are commercially available soy protein–based products which been selected on the basis of similar quantities (350 g cooked weight), form (“beef” mince and patties which can be interchanged in recipes between red meat and vegetarian groups), macronutrient composition with a focus on total protein and fat, as well as taking product availability in the local food supply into account (Table 2).

**Table 2:** Comparison of red meat and vegetarian analogue nutritional profiles.

Nutrients, per 100 g	Beef^a^	Lamb^b^	Vegetarian mince^c^	Vegetarian patties^c^
Energy (kJ)	615	551	974	950
Protein (g)	20.9	20.9	18.0	14
Fat (g)	7	5.3	12.0	14.2
Saturated fat (g)	2.5	2	8.2	8.6
Carbohydrates (g)	0	0	11.0	8.9
Sugar (g)	0	0	3.6	2.0
Sodium (mg)	47	75	368	311

^a^Beef, hindquarter sirloin, separable, lean, and raw.

^b^Lamb, hindquarter double loin, separable lean, and raw.

^c^Provided on the nutrition information panel.

Participant’s background diet can affect the physiological and psychological outcomes of interest. Efforts have been made to align participants’ diets with healthy eating guidelines and to increase consistency between intervention arms, while still providing a realistic examination of the effects of consuming moderate amounts of red meat compared rather than its vegetarian analogue. To achieve this, household units will receive a weekly vegetarian meal kit delivery (Woop Ltd) containing complete ingredients and recipe cards for cooking 3 evening meals, which will be matched across all participants. Beyond the protein and meal kit provided, participants do have a degree of choice within their diet but are provided with a cookbook and web-based nutrition support package to facilitate healthy meal choices and preparation (see *Nutrition Education*). Collectively, the objective is to ensure that participants are meeting local recommendations for servings of core food groups consumed each day (fruits/vegetables, wholegrains, protein/alternatives, and dairy/alternatives), and minimizing the intake of discretionary foods (eg, takeaways and fizzy drinks) [30].

A secondary outcome of this study is the subjective ease, compliance, and satisfaction of following a prescribed healthy eating pattern with or without red meat. This aligns with the trial objective of understanding the psychological response to following a diet with moderate amounts of red meat or vegetarian analogues.

### Nutrition Education

We have designed a web-based nutrition education package specifically for young adults, following a literature review and focus group needs assessments. The nutrition package was developed and reviewed by expert registered dietitians in the team, who are appropriately placed to ensure the accuracy and quality of information provided. The Nine Principles framework, incorporating behavioral theory and user-centered design, was used to guide its development [31]. The aim of the package is to (1) support young adults’ adoption and maintenance of healthy dietary behaviors, such as those outlined in the Eating and Activity Guidelines for New Zealand Adults [30], and (2) support adherence to respective dietary arms. Each team will have a private Facebook page and messenger chat which will be utilized to deliver evidence-based nutritional information and meal ideas. Behavior change techniques used in this study include goal-setting, behavior demonstration, and setting graded tasks. The nutrition package content and delivery will be standardized across arms to encourage consistency in the adoption of healthy dietary behaviors. Participants will only be prompted to engage with nutrition education by way of notifications through Facebook and Facebook messenger as posts are made in accordance with a predetermined schedule. eHealth use will be monitored in accordance with participant’s having viewed messages in the Facebook messenger chat, and engagement will be monitored in accordance with their “likes” and comments on Facebook messages and posts.

### Dietary Intervention Compliance

Participants will be emailed a link to register for Easy Diet Diary (Xyris Software Pty Ltd) at –t_2_. This app enables researchers to see all data entered in real time, allowing compliance to be routinely monitored during the intervention. Instructional support on using the app will be provided at –t_2_ and t_0_. From t_0_, participants are required to record all dietary intake manually through text insertion into the app 2 days a week (Sunday and Monday). For the remaining 5 days of the week (Tuesday to Saturday), participants are required to enter intake through a choice of photographs or manual text insertion to monitor participant compliance with consuming all red meat or meat analogues provided each week. Participants will receive regular text reminders to complete their diaries. Compliance to dietary recording and the dietary intervention will be monitored twice a week (every 3-4 days). Adequate dietary recording is defined as entering at least 1 full day (ie, 3 meal occasions) per 3-4–day period. If participants are noncompliant with recording or the dietary intervention within a 3-4–day period, they will be sent a reminder through SMS text message. If they are noncompliant for 3 consecutive periods, the pair will be contacted and will discontinued from the study if adherence is not improved.

### Outcomes

#### Primary Outcome Variable

- Change in concentrations of polyunsaturated fatty acids (18:2 n-6, 18:3 n-3, 20:4 n-6, 20:5 n-3, 22:5 n-3, and 22:6 n-3) in erythrocyte membranes post the intervention.

#### Secondary Outcome Variables

- Change in plasma markers of cardiovascular disease risk (total cholesterol, low-density lipoprotein [LDL] cholesterol, high-density lipoprotein [HDL] cholesterol, and triglycerides).
- Change in plasma amino acids and 1-carbon nutrients/metabolites and their posttranslational modifications.
- Change in fat-soluble vitamins (A, D, E, and K).
- Change in plasma neurotransmitters and related metabolites.
- Change in plasma inflammatory markers (tumor necrosis factor-α, interleukin [IL]-1, IL-6, IL-8, IL-10, IL-13, high-sensitivity C-reactive protein).
- Change in blood hemoglobin and iron and zinc status.
- Change in physical health status including body composition, physical activity, and muscle strength.
- Change in psychological and mental well-being, including scores on the Multidimensional Fatigue Symptom Inventory-Short Form (MSFI-SF) [32], the Depression Anxiety Stress Scales-Short Form (DASS-21) [33], the World Health Organization–Five Well-Being Index (WHO‑5) [34], and the Positive Eating Scale (PES) [35].
- Subjective experience to the diets, including eating experiences (PES), adherence to the intervention, ease of following a prescribed healthy eating pattern and satisfaction of the diet (end of intervention only).
- Adverse events.

### Outcome Measurements

#### Dietary Intake

On completion of the intervention, the data manually entered into the Easy Diet Diary will be analyzed by FoodWorks Professional (Xyris Pty Ltd), which are aligned with those in the New Zealand food composition database (New Zealand FOODfiles 2018). This will allow for analysis of the nutritional composition of participant’s diets during the intervention and to obtain insight into the dietary behaviors of participants. In addition, the Otago Food Frequency Questionnaire-Short Form will be used at t_–2_, t_5_, t_10_, and t_22_. It has been validated in New Zealand to assess overall nutrient intake over a 3-month period [36].

#### Psychological Well-being

Psychological well-being will be self-assessed at intervals during the study using the MSFI-SF [32], the DASS-21 [33], the WHO‑5 [34], and the PES [35]. Together, these scales capture well-being status related to mental health, physical vitality, flourishing, and satisfaction/pleasure in eating.

#### Physical Activity and Sleep Monitoring

All participants will wear a Huawei Band 4 Pro fitness wristband for the duration of the study. The device has an accelerometer and optical heart beat detector that will be set to continuous monitoring, and a sleep tracker which will be enabled. Participants will be asked to download the Huawei Health smartphone app (Huawei Device Co, Ltd) before the intervention. At t_5_ and t_10_ clinic visits, a weekly average number of steps and sleep duration will be collected for the previous 5 weeks from this app.

#### Body Composition

Body weight and height will be measured at each laboratory visit. Additionally, full body composition will be analyzed using dual x‑ray absorptiometry (DEXA) at t_0_ and t_10_. Each DEXA scan is 6-9 minutes long, and measures percent body fat, percent body lean mass, and bone mineral density.

#### Muscle Strength

Grip strength will be assessed with a handheld dynamometer at 3 laboratory visits.

#### Blood Erythrocyte, Plasma, and Serum Analyses

Whole blood samples will be coagulated at room temperature for 15 minutes prior to centrifugation and serum separation, which will be stored at –80°C until analysis. Erythrocytes and plasma from whole blood containing anticoagulant will be harvested and stored at −80°C until analysis.

In erythrocytes, the fatty acid composition will be analyzed using the fatty acid methyl esters assay and through lipidomics as previously described [28,37].

In plasma, glucose and cholesterol (total, LDL, HDL cholesterol) and triglycerides will be measured using a Roche Cobas c311 through the enzymatic colorimetric assay, and insulin will be measured with an electrochemiluminescence immunoassay using the Roche E411 autoanalyzer.

Free amino acids and 1-carbon metabolites will be analyzed using ultraperformance liquid chromatography in accordance with previously published methods [38,39].

Fat-soluble vitamin extraction procedures and analysis will follow a liquid chromatography–mass spectrometry (LC–MS) method [40].

Neurotransmitters and related compounds will be measured through mass spectroscopy. These include phenylethyl amine, 3,4-dihydroxyphenylalanine, dopamine, 3-methoxytyramine, 3,4-dihydroxyphenylacetic acid, homovanillic acid, norepinephrine, 3,4-dihydroxyphenylglycol, 3-methoxy-4-hydroxyphenylglycol, normetanephrine, epinephrine, metanephrine, vanillylmandelic acid, tryptophan, kynurenine, 5-hydroxytryptophan, serotonin, 5-hydroxyindoleacetic acid, α-aminobutyric acid, and γ-aminobutyric acid. The methodology utilizes a mass spectrometry probe and a stable isotope coding LC–MS method developed by AgResearch and has been optimized for plasma samples. The method is in accordance with previously published protocol data [41].

Inflammatory markers will be analyzed using the Invitrogen Inflammation 20-Plex Human ProcartaPlex Panel (catalogue number: EPX200-12185-901). Briefly, 25 µL of plasma and internal controls are incubated with magnetic beads prior to a series of wash steps. In total, 25 μL of detection antibody will be added and incubated for 30 min before adding 50 μL of Streptavidin-PE. The 96-well plate will be analyzed using the Bio-Plex 200 system (BioRad) with inflammatory markers being measured in pg/mL.

Serum iron status biomarkers (iron, unbound iron capacity, ferritin, transferrin, and soluble transferrin receptor) and whole blood hemoglobin will be analyzed using a Roche Cobas c311 by enzymatic colorimetric assay. Serum zinc concentration will be analyzed using a commercial fluorometric probe kit (Ab176725, Abcam).

#### Self-efficacy and End of Intervention Questionnaires

The self-efficacy questionnaire will be distributed at eligibility screening. The questionnaire asks participants to indicate their levels of confidence with adherence to healthy eating behaviors, adherence to the flexitarian or vegetarian food patterns, and cooking skills.

The end-of-intervention questionnaire will include closed and open-ended questions regarding the satisfaction, ease of compliance, and likelihood of continuing the food pattern in the future.

#### Adverse Events

Psychological assessments will be monitored by a psychology scientist, and those who raise concerns will be referred to counseling services, with participant permission. Serious adverse events will be reported to the Health and Disability Ethics Committee.

## Results

This study will be reported in accordance with the CONSORT (Consolidated Standards of Reporting Trials) checklist. Our results will be communicated via publication. The trial is registered with ClinicalTrial.gov (unique identifier NCT04869163).

## Discussion

### Limitations

Using a free-living, mixed meal approach has its advantages in real life applicability of study findings but may hinder the clarity in outcomes. A key aim was to compare the health effects of red meat and alternatives in combination with a healthy diet, using a clearly planned nutrition education and adherence strategy. However, we acknowledge that compliance is voluntary, and the degree to which participants comply will impact the outcomes. To address this, daily food intake will be assessed using images based or mobile phone app technology and stop-go strategies in place to best support the participants in adhering to the dietary intervention and provide confidence in participant compliance [42,43].

### Scientific and Industry Benefits

Concurrent analysis of physiological and psychological well-being provides an extraordinary opportunity to understand the broad benefits of a healthy diet. If the inclusion of pasture-raised red meat improves compliance, enjoyment, and measures of mental and physical health, then this can be communicated to markets and demographics that are important to industries in New Zealand. A sequence of scholarly publications will underpin, and be a prerequisite for, any marketing messages.

### Data Management Committee and Availability of Data and Material

The PI is responsible for project coordination and will oversee the operational aspects of the trial. A scientific advisory group will regularly monitor study implementation, as well as data generation, documentation, and reporting. The PI will communicate protocol amendments to the ethics committee and clinical trial registration. Access to data will be granted to appropriate members of the research team and to authorized representatives from the host institution to monitor or audit the study and ensure compliance with regulations. Data will be made available to external academics on reasonable request.

## Appendix

Multimedia Appendix 1 SPIRIT checklist.

Multimedia Appendix 2 Copy of informed consent form.

Multimedia Appendix 3 CONSORT-eHEALTH checklist (V 1.6.1).

## Abbreviations

CONSORT: Consolidated Standards of Reporting Trials
DASS-21: Depression Anxiety Stress Scales-Short Form
DEXA: dual x-ray absorptiometry
HDL: high-density lipoprotein
LC–MS: liquid chromatography–mass spectrometry
LCn3: long-chain omega-3 fatty acids
LDL: low-density lipoprotein
MFSI-SF: Multidimensional Fatigue Symptom Inventory-Short Form
PES: Positive Eating Scale
PI: principal investigator
SPIRIT: Standard Protocol Items: Recommendations for Interventional Trials
TFEQ-R18: Three-Factor Eating Questionnaire
WHO-5: World Health Organization–Five Well-Being Index
