# Supplementary material for: A Modern Flexitarian Dietary Intervention Incorporating Web-Based Nutrition Education in Healthy Young Adults: Protocol for a Randomized Controlled Trial
Source: JMIR Res Protoc. 2021 Dec 21;10(12):e30909. doi: 10.2196/30909 (PMC8734916; doi:10.2196/30909)
Supplement: Multimedia Appendix 1 [file resprot_v10i12e30909_app1.docx]

**APPENDIX 1: SPIRIT 2013 CHECKLIST**

Appendix 1 shows recommended items to address in a clinical trial protocol and related documents, based on the SPIRIT (Standard Protocol Items: Recommendations for Interventional Trials) checklist.

**Appendix 1**: SPIRIT 2013 Checklist

| **Section/Item** | **Item No.** | **Description** |
| --- | --- | --- |
| **Title** | 1 | A Modern Flexitarian Dietary Intervention incorporating Online Nutrition Education in Healthy Young Adults: A Protocol for a Randomised Control Trial |
| **Trial registration: ‑‑Registry identifier** | 2a | The trial is registered with ClinicalTrial.gov and the unique identifier is NCT04869163. |
| **‑‑WHO registration** | 2b | N/A |
| **Protocol version** | 3 | 3/10/2021, Version 9 |
| **Funding** | 4 | This research is funded by the Meat Industry Association Innovation Limited (a subsidiary of the New Zealand Meat Industry Association), Beef and Lamb New Zealand Limited and the New Zealand Ministry of Business, Innovation and Employment. National Science Challenge - High Value Nutrition have funded the assessment of meat consumption on inflammatory biomarkers. |
| **Roles and responsibilities:  ‑‑Authors** | 5a | Refer to Appendix 3 (Conflicts of Interest table) |
| **‑‑Trial sponsor** | 5b | Auckland UniServices, c/o The University of Auckland, Private Bag 92019, Auckland 1142, New Zealand |
| **‑‑Funders** | 5c | The funders are not involved in the collection, analysis and interpretation of data; in the writing of the report; or in the decision to submit the paper for publication as contracted. |
