# Supplementary material for: A Modern Flexitarian Dietary Intervention Incorporating Web-Based Nutrition Education in Healthy Young Adults: Protocol for a Randomized Controlled Trial
Source: JMIR Res Protoc. 2021 Dec 21;10(12):e30909. doi: 10.2196/30909 (PMC8734916; doi:10.2196/30909)
Supplement: Multimedia Appendix 2 [file resprot_v10i12e30909_app2.docx]

**APPENDIX 2: COPY OF INFORMED CONSENT FORM**

**CONSENT FORM**

**THIS FORM WILL BE HELD FOR A PERIOD OF 10 YEARS**

Project title: **Sustained well-being benefits of red meat consumption and meat-analogue meals: a 10‑week randomised clinical trial**

Principal Investigator: Dr Andrea Braakhuis (The University of Auckland)

Research Team: Dr Scott Knowles (AgResearch Ltd), Dr Emma Bermingham (AgResearch Ltd), Dr Toan Pham (The University of Auckland), Dr Rajshri Roy (The University of Auckland), Nicola Gillies (The University of Auckland), Anna Worthington (The University of Auckland) ,Dr Tamlin Conner (The University of Otago).

| - I have read the Participant Information Sheet, have understood the nature of the research and why I have been selected. I have had the opportunity to ask questions and have them answered to my satisfaction. |  |  |
| --- | --- | --- |
| - I agree to take part in this research. |  |  |
| - I have had the opportunity to use support from a family (whānau) member or a friend to help me ask questions and understand the research. |  |  |
| - I understand that I am free to withdraw participation at any time |  |  |
| - I understand that blood samples will be collected and used for research. |  |  |
| - I understand that samples will be sent around New Zealand for analysis and disposed of at the end of the study |  |  |
| - I wish for a karakia said at the time of my tissue disposal (*please circle*). | *Yes* | *No* |
| - I understand that any blood results found to be outside the normal healthy range will be conveyed to me and that if I do not wish to be informed, I cannot participate in this study. |  |  |
| - I consent to my GP or current provider being informed about any significant abnormal results obtained during the study, with my permission. | *Yes* | *No* |
| - I wish to receive the summary of findings. I understand that there may be a delay between data collection and the publication and availability of the research results (*please circle as appropriate*). | *Yes* | *No* |
| - I understand that the results from this study will be used for scientific publication and presentations. |  |  |
| - I agree not to restrict the use of any data or results that arise from this research provided such a use is only for scientific purposes. |  |  |

Name ___________________________

Signature ___________________________________________ Date ______________

Researcher’s Signature_________________________________ Date ______________

E-mail for the purposes of providing general study results:

________________________________________________________________________

APPROVED BY THE HEALTH AND DISABILITY ETHICS COMMITTEE ON 28/01/2021 Reference Number 20/STH/157
